# The electronic properties of impurities (N, C, F, Cl, and S) in Ag_3_PO_4_: A hybrid functional method study

**DOI:** 10.1038/srep12750

**Published:** 2015-08-03

**Authors:** Yang Huang, Tai Ma, Qing-yuan Chen, Chao Cao, Yao He

**Affiliations:** 1 Department of Physics, Yunnan University, Kunming 650091, China; 2 Department of Physics, Hangzhou Normal University, Hangzhou 310036, China

## Abstract

The transition energies and formation energies of N, C, F, Cl, and S as substitutional dopants in Ag_3_PO_4_ are studied using first-principles calculations based on the hybrid Hartree-Fock density functional, which correctly reproduces the band gap and thus provides the accurate defect states. Our results show that N_O_ and C_O_ act as deep acceptors, F_O_, Cl_O_, and S_P_ act as shallow donors. NO and CO have high formation energies under O-poor condition therefore they are not suitable for *p*-type doping Ag_3_PO_4_. Though F_O_, Cl_O_, and S_P_ have shallow transition energies, they have high formation energies, thus F_O_, Cl_O_, and S_P_ may be compensated by the intrinsic defects (such as Ag vacancy) and they are not possible lead to *n*-type conductivity in Ag_3_PO_4_.

Since Honda and Fujishima first discovered the photocatalytic water splitting into H_2_ and O_2_^1^, the photocatalysis of water splitting has become an active research field and a potential way to solve the severe environmental crisis and energy shortage issues^2^,^3^. TiO_2_ is the earliest photocatalyst used in water splitting, the intrinsic wide band gap of pure TiO_2_ (~3.2 eV for anatase and ~3.0 eV for rutile) confines its photon absorption to the ultraviolet (UV) region, severely limiting solar energy utilization to ~5%^4^. Metal oxides are considered as potential candidates for photoelectrochemical (PEC) water splitting because of their resistance to oxidization and possible stability in aqueous solutions^5^.

Recently, Ye *et al.* reported that cubic structure semiconductor Ag_3_PO_4_, which exhibits strong oxidation power leading to O_2_ production from water, and its quantum yield achieve up to nearly 90% under visible light^6^. This is intriguing because most photocatalysts give much poorer quantum yields of ~20%^7^. Theoretical studies have also been performed to understand their origin^7^,^8^,^9^,^10^,^11^. Reunchan and Umezawa suggest that native point defects are unlikely to be responsible for an intrinsic conductivity of Ag_3_PO_4_, which an *n*-type character was observed in the previous report, but Ag_3_PO_4_ could feasibly be doped in *n*-type fashion^7^.

First-principles density functional theory (DFT) calculations have been commonly used to study the electronic properties of point defects insulators and semiconductors. The local density approximation (LDA)^12^ or the generalized gradient approximation (GGA)^13^ functional are typically employed to describe the exchange-correlation energy within DFT. A major shortcoming of LDA and GGA calculations is the large uncertainty in the position of defect levels (and hence also formation energies) due to the severe underestimation of the semiconductor band gap. Heyd *et al.* recently proposed hybrid Hartree-Fock (HF) density functional^14^, the hybrid functional has been used to accurately reproduce the band gap of insulators and semiconductors, therefore, the use of the hybrid functional is rationalized for the description of defect physics^15^,^16^,^17^,^18^.

In this paper, we perform first-principles calculations based on the hybrid HF density functional to investigate the influence of N, C, F, Cl, and S impurities on the electronic properties of Ag_3_PO_4_. Because interstitials of these impurities usually have large formation energies, we will only consider substitutional defects. The paper is organized as follow: Details of the calculations are provided in Sec. II. The electronic properties of each impurity are described in Sec. III. Finally, Sec. IV summarizes the results.

## Methodology

The density functional calculations were performed in the Vienna *ab initio* simulation package (VASP)^19^,^20^. Interaction between the valence and core electrons was described using the projector augmented wave (PAW) approach^21^. A plane-wave basis set was used to expand the wave functions up to a kinetic energy cutoff value of 300 eV.

We used the Heyd-Scuseria-Ernzerhof hybrid functional (HSE06)^14^,^22^, which adopts a screened Coulomb potential. Hence, greatly improving the description of structural properties and band structures, including band gaps. Both of these aspects are particularly important for defects. The HSE exchange is derived from the PBE0 (Perdew-Burke-Ernzerhof (PBE) functional containing 25% exact exchange)^23^ exchange by range separation and then by elimination of counteracting long-range contributions as^4^.


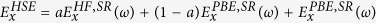


Where *a* is the mixing coefficient and *ω* is the range-separation parameter. A consistent screening parameter of *ω* = 0.2 Å^−1^ is used for the semilocal PBE exchange as well as for the screened nonlocal exchange as suggested for the HSE06 functional^24^. We find that a proportion of 33% HF exchange with 67% PBE exchange produces accurate values for lattice constants and the band gap in Ag_3_PO_4_.

We used a 128-atom supercell constructed by 2 × 2 × 2 replication of the cubic Ag_3_PO_4_ unit cell (space group 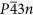), which ensures sufficient spatial separation between the periodic images of the impurities. Various dopings of Ag_3_PO_4_ have been modeled by substitution of S at P or Y (Y = N, C, F, Cl) at O sites. For geometry optimizations and electronic structure calculations, the Brillouin zone was sampled with a 2 × 2 × 2 mesh of Monkhorst-Pack special *k*-points^25^. Both the atomic positions and cell parameters were optimized until residual forces were below 0.01 eV/Å.

### Formation energies and transition levels

To determine the defect formation energies and defect transition energy levels, we follow the procedure in Ref. 26. The defect formation energy ∆*H*_f_(*α, q*) as a function of the electron Fermi energy^27^ *E*_F_ as well as the atomic chemical potentials^28^,^29^ *μ*_i_ is as follows:


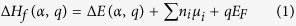


Where


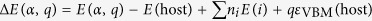


*E*(*α*, *q*) is the total energy for the studied supercell containing defect *α* in charge state *q* and *E* (host) is the total energy of the same supercell without the defect. *n*_*i*_ indicates the number of atoms of type *i* (host atoms or impurity atoms) that have been added to (*n*_*i*_ < 0) or removed from (*n*_*i*_ > 0) the supercell , and *q* is the number of electrons transferred from the supercell to the reservoirs in forming the defect cell. *E*_F_ is the electron Fermi level referenced to the valence-band maximum (VBM) of host, *ε*_VBM_(host), and varies from the valence-band maximum to the conduction-band minimum (CBM). *μ*_i_ is the chemical potential of constituent *i* referenced to its elemental solid or gas with energy *E*(*i*).

The defect transition energy level *ε*_*α*_(*q*/*q*′) is the *E*_F_ in Eq. (1), at which the formation energy ∆*H*_f_(*α, q*) of defect *α* in charge state *q* is equal to that of another charge *q*′ of the same defect, i.e.,


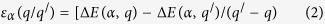


In this paper, we used a hybrid scheme to combine the advantages of both special *k*-points and Γ-point-only approaches^12^. In this scheme, for acceptor level (*q* < 0), the transition energy level with respect to VBM is given by:


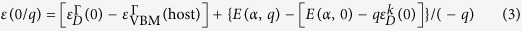


For donor level (*q* >0), the ionization energy referenced to the CBM is given by:


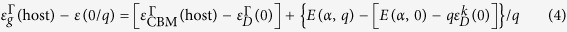


Where *ε*_D_^k^(0) and *ε*_D_^Γ^(0) are the defect levels at the special k-points (averaged) and at the Γ-point, respectively; *ε*_VBM_^Γ^(host) and *ε*_CBM_^Γ^(host) are the VBM and CBM energies, respectively, of the host at the Γ-point; and *ε*_g_^Γ^(host) is the calculated bandgap at the Γ-point.The formation energy of a charged defect is then given by


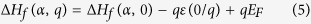


Where ∆*H*_f_(*α,* 0) is the formation energy of the charge-neutral defect. More details of calculation methods for formation energies and transition energies of defects are described elsewhere^30^.

### Chemical potentials

Under thermal equilibrium growth conditions, the steady production of host material, Ag_3_PO_4_, should satisfy the following equation:


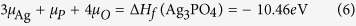


where *μ*_Ag_, *μ*_P_, and *μ*_O_ are the chemical potentials of Ag, P, and O source, respectively, and ∆*H*_f_ is the formation energy for Ag_3_PO_4_ per formula. In order to avoid the precipitation of the host elements, the chemical potential *μ*_*i*_ must be bound by


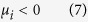


To avoid the formation of secondary phases (such as Ag_2_O and P_2_O_5_), *μ*_Ag_, *μ*_P_, and *μ*_O_ must satisfy further constrains:


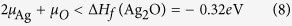



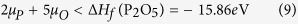


**Figure 1:**
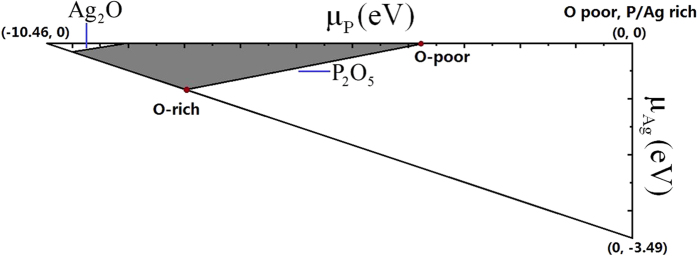
Accessible range of chemical potentials (shaded region) of equilibrium growth condition for Ag_3_PO_4_.

Considering Eqs. (6)–(9), 10, 11, 12, the accessible ranges for *μ*_Ag_, *μ*_P_, and *μ*_O_ are limited and are presented as the shaded area in Fig. 1. As shown in Eqs. (5), the calculated formation energies of charged defects depend sensitively on the selected values for *μ*_Ag_, *μ*_P_, and *μ*_O_ and the Fermi-level positions. Here, the calculated values at two representative chemical potential points are labeled as O-rich and O-poor in Fig. 1. The exact value of chemical potentials at points O-rich condition and O-poor condition are (−0.84, −7.93, 0) and (0, −3.71, −1.69) for *μ*_Ag_, *μ*_P_, and *μ*_O_, respectively.

For impurity doping, the chemical potentials of impurities also need to satisfy other constraints to avoid the formation of impurity-related phases, for example


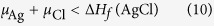



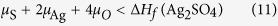



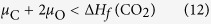



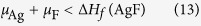


The formation enthalpies of the AgCl, Ag_2_SO_4_, AgF, and CO_2_ compounds obtained using the present HSE06 functional are listed in Table 1. The formation enthalpies obtained with the present HSE06 functional calculations are agreement with the experimental values.

## Results and Discussion

### Bulk properties

We first present the results for the structural and electronic properties of defect-free bulk Ag_3_PO_4_. The crystal structure of Ag_3_PO_4_ has a cubic structure with space group 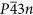, its basic structural unit is constructed by PO_4_ tetrahedron and AgO_4_ tetrahedron. The interaction between phosphorus and oxygen is mainly by covalent bond, while the interaction between silver and oxygen is formed mainly by ionic bond^8^. The optimized cell parameters are *a* = *b* = *c* = 6.02 Å, and excellent agreement with the experimental values of *a* = *b* = *c* = 6.00 Å^8^. The P-O, Ag-O, and Ag-Ag bond lengths are calculated to be 1.56 Å (experimental value^11^: 1.56 Å), 2.37 Å (experimental value^11^: 2.36 Å), 3.01 Å (experimental value^11^: 3.00 Å), respectively. The calculated indirect band gap (M−Γ) is 2.33 eV, and the direct gap at Γ is 2.45 eV, in excellent agreement with the experimental value of 2.36 eV and 2.43 eV^6^, respectively.

### N, C, F, Cl, and S impurities in Ag_3_PO_4_

**Figure 2:**
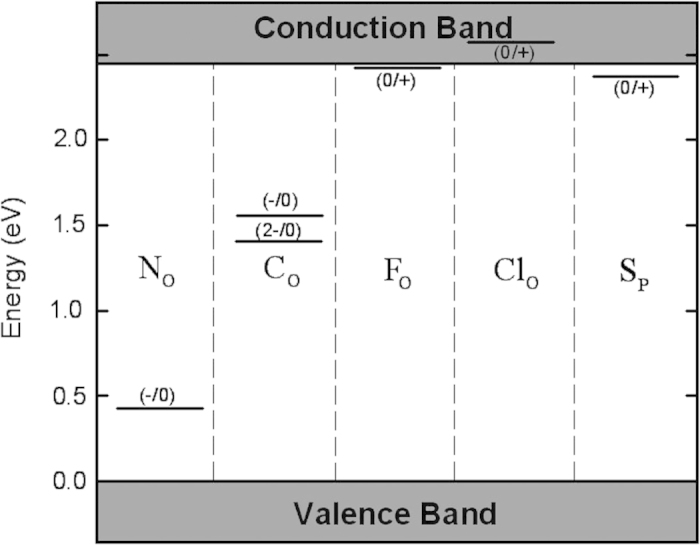
Thermodynamic transition levels for N, C, F, Cl and S impurities in Ag_3_PO_4_ (Unit: eV).

In this section, we discuss the different impurities in Ag_3_PO_4_ individually. The calculated thermodynamic transition energy levels for all impurities are listed in Fig. 2.

### Nitrogen

**Figure 3:**
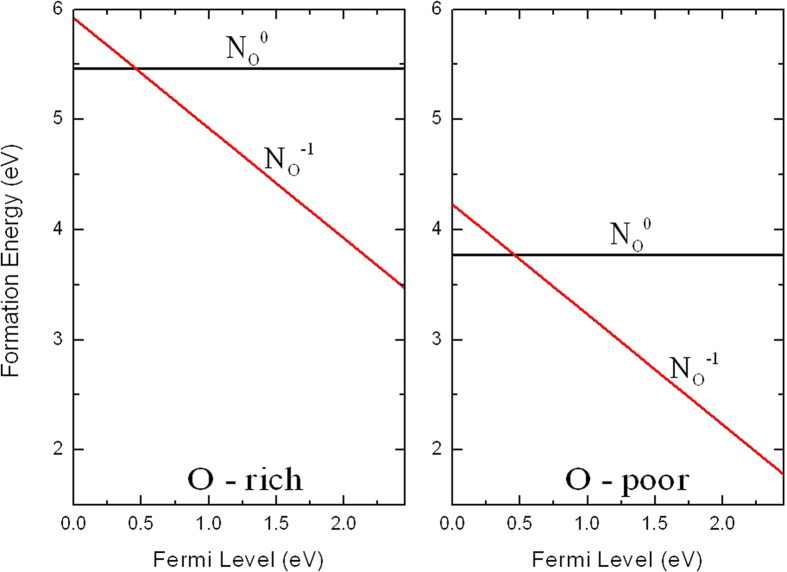
Calculated formation energies for N substituting on the O site as a function of Fermi level at O-rich and O-poor conditions.

**Figure 4:**
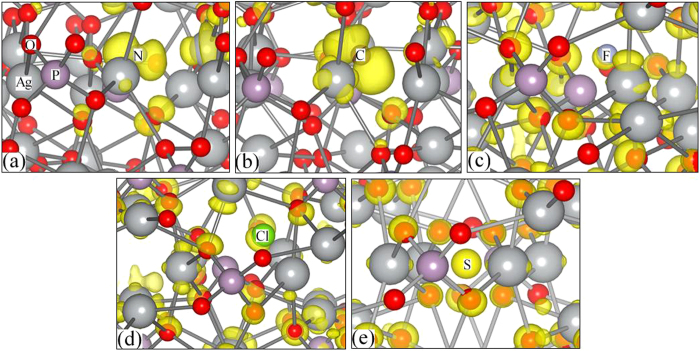
Spatial distribution of the squared wave functions 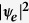 **of the neutral defect states created by** (**a**) N_O_, (**b**) C_O_, (**c**) F_O_, (**d**) Cl_O_, (**e**) S_P_, where the isosurface values^34^ are 0.05, 0.05, 0.005, 0.005, 0.005 e/Å^3^, respectively. The silver, pink, red balls represent Ag, P, O atoms, respectively.

The electronic properties of substitutional N at O sites (N_O_) has been confirmed by several reports^4^,^5^,^33^. The conductivity of a semiconductor depends not only on the thermodynamic transition levels of donor and acceptors, but also on their formation energies. The thermodynamic transition level corresponds to the intersection of the formation energies for the different charge states. Formation energy as a function of Fermi level for N_O_ in the O-rich and O-poor limit are shown in Fig. 3. When the Fermi level is low, the neutral charge state (N_O_^0^) is energetically preferable, as the Fermi level rises, above the Fermi level of 0.46 eV, the negative charge state (N_O_^−1^) becomes more favorable. The thermodynamic transition level of *ε*(−/0) is located at 0.46 eV above VBM. To obtain further details, the squared wave function 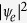
of the neutral defect state at Γ point is visualized in Fig. 4(a). The neutral defect state is highly confined around the N atom, which suggests that N_O_^0^ induces a localized defect state.

The N 2*p* orbital energy is 1.9 eV higher than the O 2*p* orbital energy, this imply that upon N substitution on O, the N 2*p* will create a partially filled impurity state at the Fermi level^35^. Thus, we examined the total density of states and projected density of states of N_O_^0^ (not shown). The neutral defect state has the main contribution from *s* orbitals and *d* orbitals of Ag, *p* orbitals of N and O, minor contribution from *p* orbitals of P. We also analyzed the local lattice relaxations around N_O_^0^ and N_O_^−1^. In the neutral state, the neighboring Ag atoms relax inward, resulting in a N-Ag bond length (2.11 Å) that is 11% shorter than the equilibrium O-Ag bond length, while the N-P bond length (1.65 Å) become 6% longer than the equilibrium O-P bond length. In the negative state, the N-Ag bond length is 2.11 Å, while the N-P bond length 1.64 Å.

Figure 3 shows that the formation energy of N_O_^0^ is more stable than N_O_^−1^ under O-poor condition but still relatively high even the Fermi level is near the conduction band. The high formation energy of the N_O_ indicates that the N-doped Ag_3_PO_4_ system using an N_2_ source may not readily to produce *p*-type conductivity, which is consistent with the N-doped ZnO system^36^.

### Carbon

As carbon atom has four valence electrons which is two electrons less than oxygen atom, the substitution of C on O site (C_O_) will act as a double acceptor. The 2*p* orbital energy of carbon is 3.8 eV higher than the O 2*p* orbital^35^. Consequently, C_O_ has two distinct transition levels in the band gap: a *ε*(−/0) transition at 1.54 eV above the VBM and a *ε*(2−/0) transition at 1.39 eV above the VBM as shown in Fig. 2. This implies that C_O_ in Ag_3_PO_4_ is a negative-*U* system. A defect often has a negative *U* if the atomic position of the defect depends sensitively on its charge state^37^, *U* refers to the additional energy upon charging of the defect with an additional electron^38^. In the neutral charge state (C_O_^0^), the three Ag nearest neighbors relax inward by 13% (of the equilibrium O-Ag bond length) while one nearest P atom slightly relaxes outward by about 13% (of the equilibrium O-P bond length).

**Figure 5:**
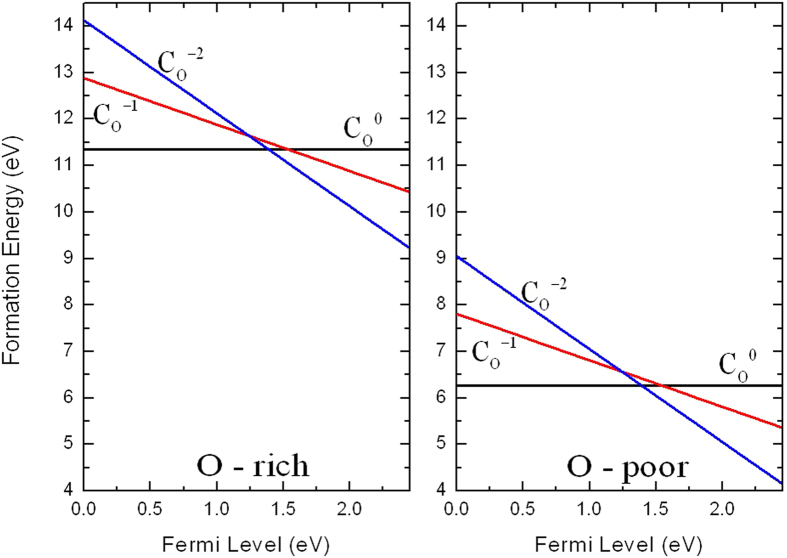
Calculated formation energies for C substituting on the O site as a function of Fermi level at O-rich and O-poor conditions.

Figure 4(b) plots the squared wave function of the neutral C_O_ defect level. One can see that the squared wave function is localized around C atom, consistent with the deep level feature. The neutral defect state has *p* orbitals of C and O, *s* orbitals and *d* orbitals of Ag contribute primarily, *p* orbitals of P contribute in a small part. The formation energies of C_O_ in the neutral, −1, and −2 charge states as a function of the Fermi level are shown in Fig. 5. Even the formation energies of C_O_ under O-poor condition is significantly lower than under O-rich condition but still relatively high, therefore C is not suitable for *p*-type doping Ag_3_PO_4_.

### Fluorine

Shifting the Fermi level towards the conduction band, or in other words *n*-type conditions, likely enhance the photocatalytic activities in Ag_3_PO_4_^7^. In contrast to the N_O_ and C_O_, the substitutional F at O site (F_O_) exhibits distinct characteristics. Unlike the N_O_ and C_O_, where the defect states are highly localized at impurities, the defect state induced by F_O_ is spatially distributed away from F as the delocalized state [see Fig. 4(c)]. The defect state has main contribution from *s* and *d* orbitals of Ag followed by *s* orbitals of F, O, and P. For F_O_^0^ the three nearest neighbor Ag atoms relax outward by 17%, while for F_O_^+1^ the relaxation are outward by 18% of the equilibrium Ag-O bond length.

**Figure 6:**
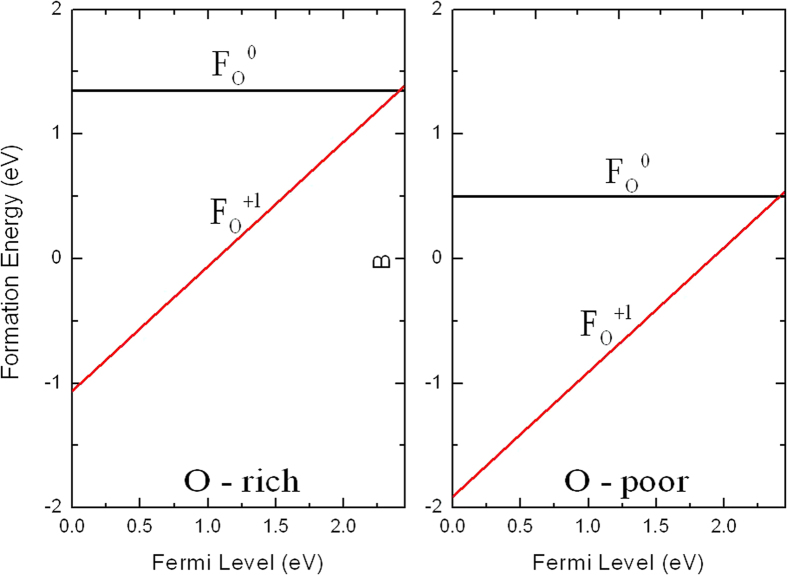
Calculated formation energies for F substituting on the O site as a function of Fermi level at O-rich and O-poor conditions.

The formation energy of F_O_ in different charge states as a function of the Fermi level is shown in Fig. 6. When the Fermi level is low, the positive charge state (F_O_^+1^) is energetically preferable, as the Fermi level rises, the formation energy approaches that of the neutral charge state (F_O_^0^), above the Fermi level of 2.41 eV, the neutral charge state becomes more favorable. The thermodynamic transition level of *ε*(0/+) is located 2.41 eV above the VBM (0.04 eV below the CBM). Though the Fermi level drops to the VBM the formation energy of F_O_ becomes very small for the favorable growth condition (O-poor condition), but when the Fermi level is near the CBM, the formation energy of F_O_ is still relatively high, thus, F_O_ may be compensated by Ag vacancy and it is not possible lead to *n*-type conductivity in Ag_3_PO_4_.

### Chlorine

**Figure 7:**
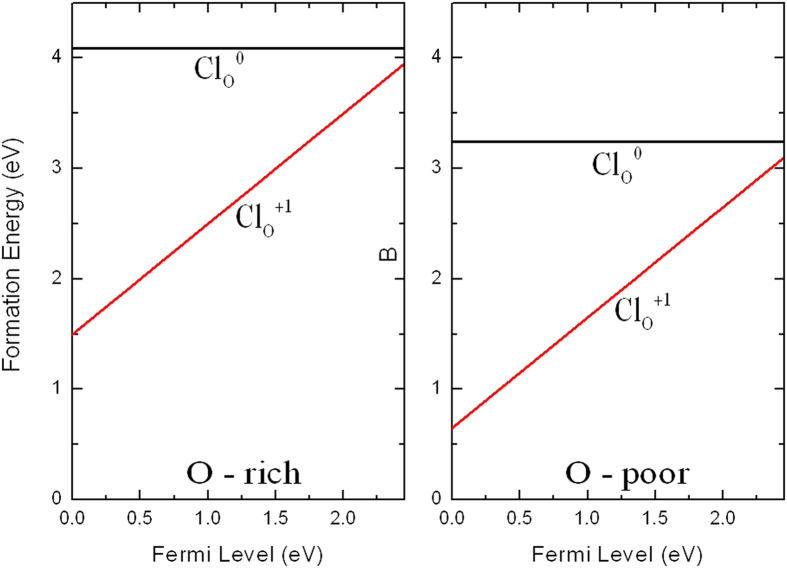
Calculated formation energies for Cl substituting on the O site as a function of Fermi level at O-rich and O-poor conditions.

Chlorine atom is a famous *n*-type dopant^37^,^39^. The formation energy of substitutional Cl (Cl_O_) against the Fermi level is shown in Fig. 7 for the two extreme cases. For Cl_O_, no transition level is find in the gap (a *ε*(0/+) transition at 0.14 eV above the CBM) and the +1 charge state is energetically favorable for the whole range of the Fermi level. The extra electron from Cl_O_^0^ occupies a conduction-band-like state, i.e., an extended state that is only slightly perturbed by the presence of the impurity^33^. Therefore, Cl_O_ is a shallow donor.

We have plotted the squared wave function of the neutral defect state at the Γ point in Fig. 4(d). It is seen that the squared wave function associated with the donor level distributed not only around Cl atom, but also around O atoms and Ag atoms away from the Cl atom, indicating a delocalized feature, which is consistent with the result that Cl_O_ is a shallow donor. The defect state has main contribution from *s* and *d* orbitals of Ag followed by *s* and *p* orbitals of Cl, O, and P. The formation energy of Cl_O_ is relatively high even under O-poor condition, therefore chlorine is not suitable for *n*-type doping Ag_3_PO_4_.

### Sulfur

Sulfur is a possible candidate for *n*-type doping when substituted for P^7^. In the case of the S substituting on the P site, the transition level *ε*(0/+) is located at 2.37 eV (0.08 eV below the CBM). The defect state is spatially away from S_P_^0^ [Fig. 4(e)], which is consistent with the result that S_P_ is a shallow donor. The defect state has main contribution from *s* and *d* orbitals of Ag followed by *s* orbitals of S, O, and P. It is also clearly from the shape of wave function that *s* and *d* orbitals are the main contributor to the defect state.

**Figure 8:**
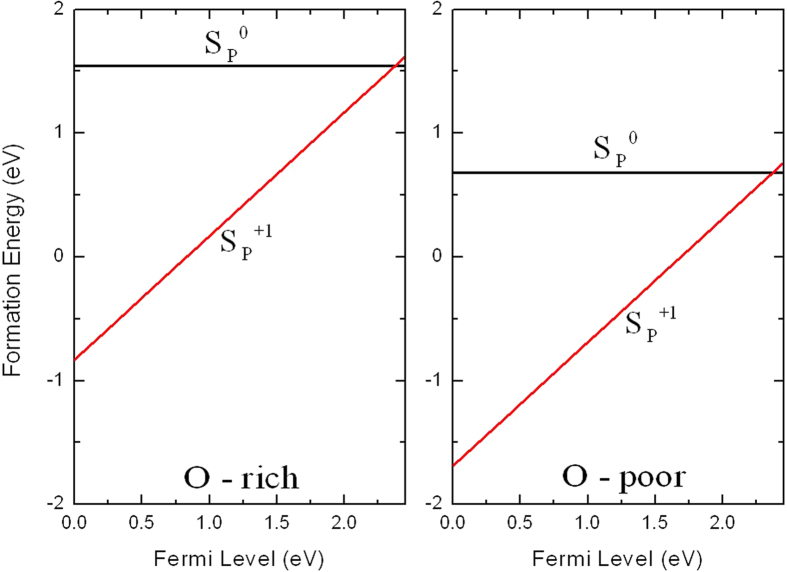
Calculated formation energies for S substituting on the P site as a function of Fermi level at O-rich and O-poor conditions.

Sulfur is surrounded by four O atoms, for S_P_^0^ these four nearest neighbor O atoms relax inward by 4% of the equilibrium P-O bond length. Formation energies of S_P_ in its various charge state are shown in Fig. 8. We note, S_P_ has lower formation energy than F_O_ and Cl_O_ for both O-rich and O-poor conditions. When the Fermi level near the VBM, S_P_ is stable in the +1 charge state. In *n*-type Ag_3_PO_4_, where the Fermi level is near the CBM, S_P_ is stable in the neutral charge state, but the formation energies in the *n*-type regime are high under O-poor condition for this sulfur is not suitable candidate for *n*-type doping Ag_3_PO_4_.

## Conclusion

Using hybrid density functional calculations we have investigated the electrical properties of N, C, F, Cl, and S impurities in Ag_3_PO_4_. We found that N_O_ and C_O_ act as deep acceptors, F_O_, Cl_O_, and S_P_ act as shallow donors. N_O_ and C_O_ have high formation energies even under most equilibrium condition (O-poor condition) therefore they are not suitable for *p*-type doping Ag_3_PO_4_. Though F_O_, Cl_O_, and S_P_ have shallow transition energies, they have high formation energies, thus F_O_, Cl_O_, and S_P_ may be compensated by Ag vacancy and they are not possible lead to *n*-type conductivity in Ag_3_PO_4_.

## Additional Information

**How to cite this article**: Huang, Y. *et al.* The electronic properties of impurities (N, C, F, Cl, and S) in Ag_3_PO_4_: A hybrid functional method study. *Sci. Rep.* **5**, 12750; doi: 10.1038/srep12750 (2015).

## Figures and Tables

**Table 1:** The formation enthalpies (in eV) of Ag_2_O, P_2_O_5_, AgCl, Ag_2_SO_4_, AgF, and CO_2_ molecules calculated from the HSE06 hybrid functional.

	HSE06	Experiment
*ΔH*_*f*_ (Ag_2_O)	−0.32	−0.32^31^
*ΔH*_*f*_(P_2_O_5_)	−15.86	−15.60^32^
*ΔH*_*f*_ (AgCl)	−1.20	−1.32^31^
*ΔH*_*f*_(Ag_2_SO_4_)	−7.06	−7.42^31^
*ΔH*_*f*_ (AgF)	−2.03	−2.13^31^
*ΔH*_*f*_(CO_2_)	−3.82	−4.08^31^

The experimental values are also listed for comparison.
